# Long-term health effects of early life exposure to tetrachloroethylene (PCE)-contaminated drinking water: a retrospective cohort study

**DOI:** 10.1186/s12940-015-0021-z

**Published:** 2015-04-12

**Authors:** Ann Aschengrau, Michael R Winter, Veronica M Vieira, Thomas F Webster, Patricia A Janulewicz, Lisa G Gallagher, Janice Weinberg, David M Ozonoff

**Affiliations:** Department of Epidemiology, Boston University School of Public Health, Talbot 3E, 715 Albany Street, Boston, MA 02118 USA; Data Coordinating Center, Boston University School of Public Health, Crosstown, 715 Albany Street, Boston, MA 02118 USA; University of California, Irvine, Program in Public Health, 653 East Peltason Dr, Irvine, CA 92697 USA; Department of Environmental Health, Boston University School of Public Health, Talbot 4 W, 715 Albany Street, Boston, MA 02118 USA; Department of Biostatistics, Boston University School of Public Health, Crosstown, 715 Albany Street, Boston, MA 02118 USA

**Keywords:** Drinking water, Tetrachloroethylene, Cancer, Epilepsy

## Abstract

**Background:**

While adult exposure to PCE is known to have toxic effects, there is little information on the long-term impact of prenatal and early childhood exposure. We undertook a retrospective cohort study to examine the effects of their early life exposure to PCE-contaminated drinking water. This retrospective cohort study examined whether prenatal and early childhood exposure to PCE-contaminated drinking water influenced the risk of a variety of chronic conditions among adults who were born between 1969 and 1983 in the Cape Cod area of Massachusetts.

**Methods:**

Eight hundred and thirty-one participants with prenatal and early childhood PCE exposure and 547 unexposed participants were studied. Individuals completed questionnaires to gather information on demographic characteristics, chronic conditions, and other sources of solvent exposure. The location of residences from birth through 1990 were used to estimate PCE exposure with U.S. EPA’s water distribution system modeling software (EPANET) modified to incorporate a leaching and transport model.

**Results:**

No associations were observed between early life PCE exposure and current occurrence of obesity, diabetes, cardiovascular disease, hypertension, color blindness, near- and far sightedness and dry eyes. In contrast, a 1.8-fold increased risk of cancer (95% CI: 0.8, 4.0) was seen among individuals with any early life exposure. These results were based on 31 participants (23 exposed and 8 unexposed) who reported cancers at a variety of anatomical sites, particularly the cervix. A 1.5-fold increase in the risk of epilepsy (95% CI: 0.6, 3.6, based on 16 exposed and 7 unexposed participants) was also observed among individuals with any early life exposure that was further increased to 1.8 (95% CI: 0.7, 4.6) among those with exposure at or above the sample median.

**Conclusions:**

These results suggest that the risk of epilepsy and certain types of cancer such as cervical cancer may be increased among adults who were exposed to PCE-contaminated drinking water exposure during gestation and early childhood. These findings should be interpreted cautiously because of the study limitations and confirmed in follow-up investigations of similarly exposed populations with medically-confirmed diagnoses. This relatively young study population should also be monitored periodically for subsequent changes in disease risk.

## Background

In 1980 Massachusetts officials found that tetrachloroethylene (PCE) was leaching into public drinking water supplies from a vinyl liner (VL) that had been applied to asbestos cement (AC) water distribution pipes [1]. The lining of vinyl toluene resin was applied in a slurry with PCE as the solvent (Piccotex; Johns-Manville Corporation, Denver, CO). The lining was introduced as a solution to taste and odor problems associated with conventional AC water mains. The PCE was thought to evaporate by the time the pipes were installed; however, sizeable quantities remained and slowly leached into the drinking water supplies.

Surveys estimated that approximately 660 miles of VL/AC pipes were installed in nearly 100 cities and towns in Massachusetts from 1968 to 1980. Because the pipes were installed in response to replacement and expansion needs in a town’s water system, the contamination pattern was irregular and neighboring residents often had vastly different exposure levels. PCE levels in one town ranged from undetectable to 80 μg/L in water pipes along main streets with high water flow and from 1,600 to 7,750 μg/L in water pipes along dead end streets with low water flow [2].

This specific and unusual source of contamination has since been brought down to what US EPA considers acceptable levels by flushing and bleeding water from the affected pipes, but PCE contamination of public water supplies is common, with adverse health consequences attributed to public water sources contaminated by PCE from landfills and leaking underground storage tanks [3], and dry cleaning facilities [4]. Thus, community-based PCE exposure remains an important topic of investigation.

Exposure to PCE from drinking water occurs through direct ingestion, dermal exposure during bathing and, because it volatilizes easily, by inhalation during showering, bathing and other household uses. According to the US EPA, PCE is considered “likely to be carcinogenic in humans” by all exposure routes, and to have toxic effects on the central nervous, renal, digestive, immune and hematological systems [5,6]. These conclusions were based mainly on animal experiments and human studies of adults exposed in occupational and environmental settings. Few studies have investigated possible health effects among individuals exposed during gestation and early childhood, and most of these studies have examined only health effects during childhood [7-10].

For these reasons, we undertook a population-based retrospective cohort study to determine if prenatal and early childhood exposure to PCE contaminated drinking water had a long-term impact on health. Outcomes previously reported in our study population included mental illness [11], affinity for risky behaviors [12], visual deficits in color discrimination and contrast sensitivity [13], and performance on neuropsychological tests [14]. The current report describes the impact of early life exposure on the risk of cancer, obesity and chronic diseases of the central nervous, visual, cardiovascular, endocrine, and musculoskeletal systems.

## Methods

### Selection of study population

Individuals were eligible for the present study if they were born between 1969 and 1983 to married women living in one of eight Cape Cod, Massachusetts towns known to have some VL/AC pipes in their water distribution system [1]. The exclusion of unmarried women and their children was an approval requirement of the Massachusetts Department of Public Health Privacy and Data Access Office. Eligible individuals were identified by reviewing birth certificates and cross-matching the maternal address and date of birth on the certificate with information collected from water companies on the location and installation year of VL/AC pipes.

To efficiently identify subjects who were likely to be exposed or unexposed, we visually inspected maps depicting the pipe distribution network in the vicinity of the birth address. Subjects were tentatively designated as “exposed” when their birth residence was either directly adjacent to a VL/AC pipe or indirectly adjacent to a pipe connected to a VL/AC pipe with the only possible water flow through VL/AC pipe (N = 1,910). Subjects who were initially designated as “unexposed” were randomly selected from the remaining resident births during this time period and frequency matched to exposed subjects on month and year of birth (N = 1,928).

In addition, 1,202 older siblings of exposed and unexposed subjects were identified if they were born in Massachusetts during 1969-1983. All older siblings were initially considered unexposed during the prenatal period because they were born while the family lived at an apparently unaffected residence. However, the initial exposure status of all subjects was considered tentative until more extensive exposure assessments were completed, as described below.

Birth certificates were reviewed to obtain information on the family, including the full names of the subject and parents; the subject’s date of birth, birth weight and gestational duration; and the parents’ ages and educational levels at the subject’s birth. The study was approved by the Institutional Review Boards of the Massachusetts Department of Public Health and Boston University Medical Center and by the 24A/B/11B Review Committee at the Massachusetts Department of Public Health.

### Follow-up and enrollment

Follow-up and enrollment occurred during 2006-2008. As described in Table 1, 6.6% of the selected population could not be located (n = 332), 45.5% were located but never responded to our contact attempts (n = 2,294), 3.7% refused to participate (n = 187), and 2.2% were deceased (n = 111). The Massachusetts Department of Public Health IRB did not allow us to contact another 8.5% of subjects whose parent had refused to participate in our prior cohort study of reproductive and developmental outcomes (n = 427) [15]. These percentages were similar for both exposed and unexposed subjects and their older siblings.

**Table 1:** **Selection, enrollment, and initial and final exposure status of study subjects**

	**Initial exposure status**			
	**Index subject**		**Older sibling**	
	**Exposed**	**Unexposed**	**Unexposed**	**Total**
Selected	1910	1928	1202	5040
Excluded during enrollment				
Deceased	35	40	36	111
Parent refused participation	199	148	80	427
Never located	113	149	70	332
No response	871	887	536	2294
Refused	73	78	36	187
Returned questionnaire	619	626	444	1689
Percent of selected	32.4%	32.5%	36.9%	33.5%
Percent of located	39.6%	39.3%	43.7%	40.5%
Excluded during exposure assessment				
Inadequate residential history	15	37	29	81
Off-Cape address in potential	19	27	50	96
VL/AC town				
Available for analysis	585	562	365	1512
Percent of selected	30.6%	29.1%	30.4%	30.0%
Final exposure status				
Both prenatal and early	561	160	110	831
childhood exposure				
Only early childhood	7	42	85	134
Exposure				
Unexposed	17	360	170	547

Available birth certificate and water distribution data also indicated that participants and non-participants were similar with respect to their initial PCE exposure status (36.7% and 38.8% of participants and non-participants were considered exposed), race (98.5% and 97.3%, respectively, were White), mean age (29.3 and 28.9 years, respectively, as of June 30, 2007), mean duration of gestation (40.1 and 39.9 weeks, respectively), and mean birth weight (3,428 and 3,401 grams, respectively). Participants were more likely to be female (60.7% of participants vs. 43.5% of non-participants), and have mothers with some college education (61.0% of participants vs. 49.3% of non-participants); however, these differences were present for both exposed and unexposed non-participants.

A self-administered questionnaire was sent to all successfully traced subjects to gather information on height and weight (to estimate body mass index), and chronic conditions, including diagnoses of cancer; diabetes; hypertension; cardiovascular disease; epilepsy and seizure disorders; stroke, mini stroke and transient ischemic attack; multiple sclerosis; lupus; amyotrophic lateral sclerosis; Huntington’s disease; Parkinson’s disease; rheumatoid arthritis; scleroderma; and vision impairment resulting from blindness, color blindness, glaucoma, macular degeneration, cataracts, far sightedness and near sightedness. All disease-related questions asked if a doctor or health care provider had ever stated that the participant had a particular condition and what year the condition was diagnosed. Respondents were also asked to rate their own overall health (i.e., excellent, very good, good, fair and poor) and in comparison to other people their own age (i.e., better than most, about the same, and worse than most).

Information was also gathered on demographic characteristics; family history of cancer and other relevant diseases; health-related behaviors such as cigarette smoking, alcoholic beverage consumption and illicit drug use; occupational and non-occupational exposure to solvents; and residential addresses from birth through 1990. Lastly, the survey gathered information on the subject’s knowledge of the PCE contamination episode and self-assessments of PCE exposure. Additional questionnaire data on maternal characteristics and behaviors during the prenatal period and certain health outcomes (e.g. cancer) were available for subjects whose mothers participated in our prior cohort study on the impact of PCE exposure on reproduction and development. Information on the mother’s water use patterns was available for only 58% of subjects and the prevalence of key variables was relatively low (e.g., bottled water use) and so these data were omitted from our analyses.

### Geocoding of residential addresses

Approximately 95% of reported addresses were successfully geocoded using ArcGIS 8, and all geocoding was conducted without knowledge of the exposure or outcome status. In most instances, each address was geocoded to a parcel of land. Addresses that could not be geocoded to a specific parcel were geocoded to the closest parcel by street number. If a street number was unavailable and the street was less than a mile long, the address was geocoded to the middle of the street. If the street was a mile or longer, the address was geocoded to the intersection of the street with the cross-street provided in the survey.

### PCE exposure assessment

As previously described, a tentative exposure status was assigned to each subject by visually inspecting maps of the pipe distribution network in the area surrounding the birth residence. To determine the final exposure designation, we used a more extensive exposure assessment model to estimate the mass of PCE delivered to each residence from the prenatal period through five years of age.

#### The Webler-Brown Model and EPANET

The leaching and transport model, which was developed by Webler and Brown for our prior epidemiological studies [16,17], estimates the quantity of PCE entering the drinking water using the initial amount of PCE in the liner (based on the pipe diameter and length), the age of the pipe, and the leaching rate of PCE from the liner into the water. Information on the locations, installation dates and diameters of all VL/AC pipes in the public water supplies was provided by local water departments and the Massachusetts Department of Environmental Protection (DEP). According to by laboratory experiments by Demond, the leaching process followed a simple exponential relationship [2].

The transport algorithm requires an estimate of water flow rate and direction, which are functions of the configuration of the pipes and number of water users. The present study incorporated the Webler and Brown algorithm into the publically available source code of EPANET water distribution modeling software which characterizes water flow throughout a town’s entire public distribution system [18]. EPANET, which was developed by the US EPA for water monitoring programs, has been also applied in epidemiological studies by others to assess the health effects of drinking water contaminants [19,20].

#### GIS together with the models

In the first step of the exposure assessment process, we created GIS layers depicting the subject residences, water sources, pipe characteristics, and nodes, that represented points of water consumption along the pipe. Data on the location, installation date, and diameter of VL/AC pipes was obtained from local water companies and the Massachusetts Department of Environmental Protection. The GIS represented the pipe configuration in the period around 1980.

Next, we used EPANET to simulate the instantaneous flow of water through each town’s network and to estimate the annual mass of PCE delivered to each point on the network or node and all subject residences associated with the node. We assumed that all land parcels represented water users, all water users in the network drew the same quantity of water, and water sources did not change over the study period. These assumptions are supported by observations that the study area was mainly comprised of residences, and the distribution system changed little between the late 1960s and late 1980s, when some water sources were added to accommodate population growth.

Only annual PCE exposures were calculated because only move-in and pipe installation years were available. We estimated PCE exposure during the prenatal period by multiplying the annual mass of PCE that entered the subject’s residence during their birth year by 9/12. We estimated cumulative exposure during early childhood by summing the estimated mass of PCE that entered their residences from the month and year following birth through the month and year of the fifth birthday. Simple proportions were used to account for partial years.

PCE exposure levels were estimated only for subjects who had complete geocoded residential histories from birth through age five. This excluded 81subjects because they had inadequate residential histories (Table 1). For practical reasons, another 96 subjects were excluded because at least one of their residences was in an off-Cape town with some VL/AC pipe and our extensive PCE exposure assessments were limited to Cape Cod. Subjects who reported living in a Cape Cod town without any VL/AC pipes (n = 7) were assumed to have no PCE exposure at that address because available records indicated little or no PCE contamination of these water sources.

### Statistical analysis

We compared the occurrence of each chronic condition among subjects with combined prenatal and early childhood exposure to unexposed subjects. First, we examined the impact of any PCE and then divided the exposure at the median to examine whether risk was related to higher or lower level of exposure. Nearly all subjects with prenatal exposure also had childhood exposure and so we were unable to examine the impact of prenatal exposure only. In addition, we did not examine the impact of exposure only during childhood because there were too few subjects in this category (n = 134) to provide stable effect estimates of the outcomes under investigation.

The risk ratio (RR) was used to estimate the strength of the association between PCE exposure and the occurrence of each chronic condition. Ninety-five percent confidence intervals were used to assess the precision of the risk ratios. Generalized estimating equation (GEE) analyses were performed to account for non-independent outcomes arising from several children from the same family [21,22]. The log link was used while assuming equal correlation between birth outcomes from the same mother. These analyses were performed only if there were at least five exposed and five unexposed participants who reported a particular health outcome.

Adjusted GEE analyses were conducted to assess the influence of confounding. Variables considered for these analyses were demographic, medical and family characteristics, and non-drinking water sources of solvent exposure. These variables included the subject’s gender, race, age, educational level, employment status, and marital status; cigarette smoking, alcoholic beverage consumption and illicit drug use; history of solvent-related jobs and hobbies; family history of cancer and other diseases; parental characteristics and behaviors during the subject’s pregnancy, and a history of head trauma (for analyses of epilepsy). Each of these variables was added to the GEE model one at a time to assess the presence of confounding. Most of the potential confounding variables had very little missing data and so individuals with missing data were dropped from these analyses. Only age had a meaningful impact (>10% change) in the crude estimates of associations and so this was the only variable included in the final adjusted GEE model.

## Results

A total of 1,512 subjects were available for the analysis. According to the initial exposure designation, there were 585 exposed and 562 unexposed index subjects and 365 unexposed older siblings (Table 1). Following the more refined exposure assessment, approximately 27.4% of subjects (N = 414) changed their status during the detailed exposure assessment either because (1) their birth address was further downstream from a VL/AC pipe than was considered exposed in the preliminary visual assessment, (2) their modeled assessment indicated a different water flow direction than was originally assumed, (3) their questionnaire data indicated that they used a private wells, or (4) their questionnaire data indicated that they lived in an affected residence during childhood. Thus, the final analytic groups were comprised of 831 subjects with prenatal and early childhood exposure and 547 subjects with no exposure during either period. As previously mentioned, 134 subjects with only childhood exposure were excluded from the analysis.

As shown in Table 2, the characteristics of the exposed and unexposed subjects were quite similar. Subjects were predominantly female, white, college-educated, in their late 20s, married or cohabitating, and employed when they completed the study questionnaires. Few subjects had possible occupational exposure to solvents but many had potential exposure from hobbies and other sources. Family history of relevant illnesses, parental characteristics, and the frequency of cigarette smoking, and alcoholic beverage consumption was also similar across groups; however, the frequency of illicit drug use (e.g., crack, cocaine, psychedelics/hallucinogens, club/designer drugs, Ritalin without a prescription, and heroin) was more common among exposed participants [14].

**Table 2:** **Distribution of selected characteristics of subjects and parents by PCE exposure status**

**Characteristic**	**Prenatal and early childhood exposure**		**Unexposed**	
	**(N = 831)**		**(N = 547)**	
	**n**	**%**	**n**	**%**
Current age (n, mean, sd)	831	29.2 (3.6)	547	29.6 (3.8)
Gender				
Male	331	39.8	216	39.5
Female	500	60.2	331	60.5
% White race	818	98.4	539	98.5
Current educational level				
High school graduate or less	128	15.4	67	12.2
Some college	192	23.1	144	26.3
Four year college grad or higher	510	61.4	335	61.2
Missing	1	0.1	1	0.2
Currently employed				
Yes	719	86.5	487	89.0
No	92	11.1	54	9.9
Missing	20	2.4	6	1.1
Current marital status				
Single	272	32.7	157	28.7
Married or cohabitating	536	64.5	371	67.8
Other	19	2.3	12	2.2
Missing	4	0.5	7	1.3
Ever had solvent-exposed job				
Yes	123	14.8	71	13.0
No	687	82.7	461	84.3
Missing	21	2.5	15	2.7
Ever had solvent-exposed hobby				
Yes	700	84.2	462	84.5
No	124	14.9	79	14.4
Missing	7	0.8	6	1.1
Cigarette smoking history				
Current	194	23.3	104	19.0
Former	180	21.7	134	24.5
Never	451	54.3	308	56.3
Missing	6	0.7	1	0.2
Current alcoholic beverage consumption				
>8 days per month	256	30.8	181	33.1
<= 8 days per month	413	49.7	255	46.6
Non-drinker	149	17.9	108	19.7
Missing	13	1.6	3	0.5
Current major^a^ drug use				
Yes	289	34.8	162	29.6
No	530	63.8	380	69.5
Missing	12	1.4	5	0.9
Mother’s age^b^ (n, mean (sd))	831	27.2 (4.7)	547	27.5 (4.4)
Father’s age^b^ (n, mean (sd))	831	29.8 (5.7)	547	29.8 (5.3)
Mother’s educational level^b^				
High school graduate or less	327	39.4	178	32.5
Some college	243	29.2	188	34.4
Four year college grad or higher	260	31.3	180	32.9
Missing	1	0.1	1	0.2
Father’s occupation^b^				
White collar	420	50.5	257	47.0
Blue collar	275	33.1	170	31.1
Other	126	15.2	112	20.5
Missing	10	1.2	8	1.5
Mother received prenatal care^b^				
Yes	794	95.5	520	95.1
No	4	0.5	0	0.0
Missing	33	4.0	27	4.9
Mother smoked cigarettes^b^				
Yes	182	21.9	113	20.7
No	483	58.1	330	60.3
Missing	166	20.0	104	19.0
Mother drank alcoholic beverages^b^				
Yes	302	36.3	201	36.7
No	361	43.4	242	44.2
Missing	168	20.2	104	19.0
Mother had medical and obstetrical complications^b^				
Yes	122	14.7	108	19.7
No	536	64.5	331	60.5
Missing	173	20.8	108	19.7
Mother had occupational exposure to solvents				
Yes	76	9.1	51	9.3
No	573	69.0	381	69.7
Missing	182	21.9	115	21.0
Subject’s birth weight (n, mean, (sd))	823	3,443 (506)	499	3,414 (534)
Subject’s gestational age (n, mean, (sd))	790	40.1 (2.5)	516	39.9 (2.4)
Family history of cancer	225	27.1	141	25.8
Family history of epilepsy or seizure disorder	39	4.7	23	4.2
Family history of rheumatoid arthritis	64	7.7	41	7.5
Family history of blindness	11	1.3	6	1.1
Family history of color blindness	64	7.7	45	8.2
Family history of dry eyes	60	7.2	39	7.1
Family history of correctable^c^ vision impairment	786	94.6	514	94.0

^a^Major drugs include inhalants, cocaine, crack, psychedelics, hallucinogens, club drugs, designer drugs, heroin, and Ritalin without a prescription.

^b^At subject’s birth or during subject’s gestation.

^c^Correctable with eyeglasses or contact lenses.

The study population was exposed to a wide distribution of PCE exposure levels that covered several orders of magnitude. Cumulative prenatal exposure levels were lower than early childhood levels because of their different durations (nine months vs. five years). Mean (SD) exposures were 32.6 (88.6), 109.0 (283.3), and 141.6 (358.1) grams, respectively, for prenatal, early childhood and combined prenatal and early childhood exposure.

When we compared each subject’s self-assessed exposure status to that derived from the modeled assessment, we found that only 7% of subjects considered exposed by the modeled assessment thought that their drinking water was contaminated, whereas 29% of these exposed subjects thought that their water was not contaminated and 64% were unsure. Similarly, we found that 31% of subjects considered unexposed by the modeled assessment thought that their drinking water was not contaminated while 5% thought that their drinking water was contaminated and 63% were unsure.

The relationship between early life PCE exposure and self-reported health outcomes is described below and summarized in Table 3. Compared to unexposed subjects, twice as many exposed subjects considered themselves to be in fair or poor health (4.5% versus 2.2%) and nearly three times as many exposed subjects considered their health to be worse than most people their own age (7.1% versus 2.6%).

**Table 3:** **Prenatal and early childhood exposure to tetrachloroethylene and the risk of chronic health conditions**

**Health outcome**	**Exposure**	**% Yes (n/N)**	**Crude GEE**	**Adjusted GEE**
	**Category/**		**Risk ratio**	**Risk ratio**
	**Percentile**		**(95% CI)**	**(95% CI)**
Cancer	Any	2.8 (23/828)	1.9 (0.9, 4.2)	1.8 (0.8, 4.0)
	> = 50^th^	2.9 (12/415)	2.0 (0.8, 4.8)	1.7 (0.7, 4.5)
	>0- < 50^th^	2.7 (11/413)	1.8 (0.7, 4.5)	1.9 (0.7, 4.8)
	None	1.5 (8/545)	Reference	Reference
Diabetes	Any	1.2 (10/828)	0.9 (0.4, 2.5)	1.0 (0.4, 2.6)
	> = 50^th^	1.2 (5/415)	0.9 (0.3, 2.9)	0.9 (0.3, 2.7)
	>0- < 50^th^	1.2 (5/413)	0.9 (0.3, 2.9)	1.1 (0.3, 4.2)
	None	1.3 (7/544)	Reference	Reference
Hypertension	Any	8.5 (70/824)	1.2 (0.8, 1.8)	1.2 (0.8, 1.8)
	> = 50^th^	8.2 (34/414)	1.2(0.8, 1.8)	1.1 (0.7, 1.7)
	>0- < 50^th^	8.8 (36/410)	1.3 (0.8, 1.9)	1.4 (0.9, 2.2)
	None	7.0 (38/543)	Reference	Reference
Cardiovascular Disease	Any	0.6 (5/825)	0.4 (0.1, 1.2)	1.5 (0.6, 3.6)
	> = 50^th^	0.5 (2/414)	-----	-----
	>0- < 50^th^	0.7 (3/411)	-----	-----
	None	1.5 (8/541)	Reference	Reference
Epilepsy	Any	1.9 (16/826)	1.5 (0.6, 3.6)	1.5 (0.6, 3.6)
	> = 50^th^	2.2 (9/415)	1.7 (0.6, 4.5)	1.8 (0.7, 4.6)
	>0- < 50^th^	1.7 (7/411)	1.3 (0.5, 3.7)	1.2 (0.4, 3.5)
	None	1.3 (7/543)	Reference	Reference
Obesity	Any	24.7 (138/558)	0.9 (0.7, 1.2)	0.9 (0.7, 1.2)
	> = 50^th^	26.0 (71/273)	1.0 (0.7, 1.3)	1.0 (0.7, 1.3)
	>0- < 50^th^	23.5 (67/285)	0.9 (0.7-1.2)	0.9 (0.7, 1.2)
	None	26.7 (92/344)	Reference	Reference
Color blindness	Any	1.4 (11/779)	0.6 (0.3, 1.3)	0.6 (0.3, 1.3)
	> = 50^th^	1.0 (4/386)	-----	-----
	>0- < 50^th^	1.8 (7/393)	0.7 (0.3, 1.8)	0.8 (0.3, 2.2)
	None	2.5 (13/528)	Reference	Reference
Near sightedness	Any	22.1 (171/775)	1.0 (0.8, 1.2)	1.0 (0.8, 1.2)
	> = 50^th^	21.6 (83/385)	1.0 (0.7, 1.2)	0.9 (0.7, 1.2)
	>0- < 50^th^	22.6 (88/390)	1.0 (0.8, 1.3)	1.0 (0.8, 1.3)
	None	22.6 (118/523)	Reference	Reference
Far sightedness	Any	55.6 (429/771)	1.0 (0.9, 1.1)	1.0 (0.9, 1.1)
	> = 50^th^	56.4 (215/381)	1.0 (0.9, 1.1)	0.9 (0.8, 1.1)
	>0- < 50^th^	54.9 (214/390)	0.9 (0.8, 1.1)	1.0 (0.9, 1.1)
	None	58.3 (307/527)	Reference	Reference
Dry eyes	Any	8.4 (65/775)	1.2 (0.8, 1.8)	1.2 (0.8, 1.8)
	> = 50^th^	8.8 (34/386)	1.3 (0.8, 2.0)	1.2 (0.8, 1.9)
	>0- < 50^th^	8.0 (31/389)	1.1 (0.7, 1.8)	1.3 (0.8, 2.0)
	None	7.0 (37/528)	Reference	Reference

In line with these self-assessments, individuals with early life PCE exposure had a 1.8-fold increased risk of cancer (95% CI: 0.8, 4.0). This finding was based on 31 cancer cases (23 exposed and 8 unexposed) who reported a wide variety of cancers diagnosed between ages 4 and 32 years (median age at diagnosis = 25 years) (Table 4). Notably, a history of early life PCE exposure was present for all five reported cases of cervical cancer, both reported cases of brain cancer, and eleven of the thirteen cases who reported a wide variety skin cancer cases (including melanoma, basal cell carcinoma, squamous cell carcinoma and skin cancer not otherwise specified). A 2.8-fold increased risk of all skin cancers combined was observed in relation to early life PCE exposure (95% CI: 0.6, 13.0). Risk ratios for other cancer sites were not estimated because of the small number of cases.

**Table 4:** **Cancer diagnoses among exposed and unexposed participants**

**Cancer site/type**	**Total number**	**Number with any exposure**	**Number unexposed**
Cervix	5	5	0
Ovary	2	1	1
Testes	2	0	2
Hodgkin lymphoma	1	1	0
Lymphoma, not otherwise specified	1	1	0
Leukemia	1	1	0
Brain	2	2	0
Thyroid	2	0	2
Pancreas	1	1	0
Sarcoma	1	0	1
Melanoma	4	4	0
Other skin cancer (basal or squamous)	7	5	2
Skin cancer, not otherwise specified	2	2	0
Total	31	23	8

Regarding the non-cancer outcomes, individuals with any early life PCE exposure also had a 1.5-fold increased risk of epilepsy (95% CI: 0.6, 3.6) that was further increased to 1.8 (95% CI: 0.7, 4.6) among individuals with exposure at or above the sample median (Table 3). The age of diagnosis among the epilepsy cases ranged from infancy to 24 years (median age at diagnosis = 17.5 years). In contrast, no associations were observed between early life PCE exposure and the occurrence of obesity, diabetes, cardiovascular disease, hypertension, color blindness, near- and far sightedness and dry eyes. The frequency of the remaining illnesses was too rare to calculate stable measures of association: stroke, mini stroke or transient ischemic attack (n = 6 overall); lupus (n = 3); multiple sclerosis (n = 5); amyotrophic lateral sclerosis (n = 1), scleroderma (n = 0), Huntington’s disease (n = 0), Parkinson’s disease (n = 0), rheumatoid arthritis (n = 15), deafness (n = 6), blindness (n = 12), cataracts (n = 6), glaucoma (n = 1), and macular degeneration (n = 2).

## Discussion

While the number of cases was small, the results of this study suggest that individuals with early life exposure to PCE-contaminated drinking water may have an increased risk of epilepsy and certain types of cancer, particularly cervical cancer. No increases in risk were observed for obesity, diabetes, cardiovascular disease, hypertension, and clinically evident vision abnormalities.

These findings should be interpreted cautiously in light of several limitations. First, the young age of the participants (mean ages were 29.6 and 29.2 years for exposed and unexposed subjects, respectively) meant that the frequency of many chronic conditions was low, including cancers linked to PCE exposure in previous occupational and drinking water studies [5]. While the prevalence of epilepsy, diabetes and other conditions was similar to national estimates for this age group [23,24], it is likely that the use of self-reported information on health outcomes resulted in some inaccurate information stemming from under-reporting, over-reporting or mis-reporting diagnoses. However, since most subjects did not know their exposure status we believe that the resulting outcome misclassification would not have affected the risk ratios or would have biased them towards the null [25].

The findings are also likely affected by exposure misclassification. Because historical exposure measurements were unavailable, we estimated PCE exposure using a leaching and transport model [16,17]. The model was applied to water distribution system conditions in 1980 and was assumed to be representative of the entire exposure period. Furthermore, the exposure assessment predicted the annual mass of PCE delivered to each subject’s residence during gestation and early childhood. Because information on maternal water use was available for only 58% of the study population, we did not incorporate these behavioral data into our analyses. While results from validation studies indicate reasonable correlation between our exposure estimates and PCE concentrations in historical water samples (Spearman correlation coefficient = 0.65, p < 0.00010) [26], non-differential exposure misclassification likely biased the findings from dichotomous comparisons (e.g., any exposure vs. none) towards the null [27]. The expected direction of bias for comparisons involving the exposure levels is more difficult to predict but associations seen among subjects in the highest tertile are likely to be attenuated while those in the middle category could have either an upward or downward bias.

Still another limitation stems from possible residual confounding from missing data on risk factors for the outcomes under study. However, in order to account for the associations observed in this study, these factors would need to be tightly correlated with PCE exposure, an unlikely scenario given the irregular pattern of the PCE contamination across the neighborhoods of Cape Cod. In fact, our prior studies of this cohort found little or no confounding when other health outcomes were examined [15,28].

A further limitation stems from the study’s low response rate. Although this problem reduced the statistical power of the study, the following evidence suggests that it did not result in selection bias. First, most available characteristics of participants and non-participants were similar, including initial PCE exposure status, race, mother’s age at delivery, and birth characteristics. Second, while participants were more likely to be female (60.7% of participants vs. 43.5% of non-participants), and have mothers with some college education (61.0% for participants vs. 49.3% for non-participants), these differences were equally present for exposed and unexposed non-participants. Third, losses stemming from the death of potential participants were small and unrelated to initial PCE exposure status (n = 111, Table 1).

PCE’s potential to cause cancer has been established through numerous animal and human studies that have contributed to PCE’s designation as a “probable human carcinogen” by the International Agency for Cancer Research (IARC) and “likely to be carcinogenic in humans” by the US EPA [5,6,29]. Evidence comes from animal experiments where increases in mononuclear cell leukemia and kidney tumors among PCE-exposed rats and hepatocellular tumors, hemangiomas and hemangiosarcomas among PCE-exposed mice have been observed [6]. Evidence from human studies comes mainly from investigations of dry cleaners and other adults occupationally exposed to PCE and related solvents [30-32]. However, studies of adult exposure to PCE-contaminated drinking water have also contributed to the literature [33,34]. Taken together, epidemiological studies provide evidence for associations between adult PCE exposure and bladder cancer, non-Hodgkin lymphoma and multiple myeloma, and possibly cervical, esophageal, kidney, lung, liver and breast cancer [5,6]. To the best of our knowledge, there are little data on the risk of skin cancer following adult PCE exposure.

In contrast, the few epidemiological studies on the risk of cancer following early life exposure to solvents such as PCE have produced mixed results. One study found a null association between the occurrence of neuroblastoma among offspring and occupational exposure to PCE among mothers [35]. In contrast, three studies of childhood hematopoietic cancers have found positive associations. The largest of these studies found a 1.6-fold increased risk of acute lymphocytic leukemia (95% CI:1.1-2.3) among children whose mothers worked with a variety of solvents during pregnancy [36]. Two additional small studies also found positive associations between prenatal exposure to drinking water contaminated with PCE, TCE and other solvents and either childhood leukemia alone or in combination with non Hodgkin lymphoma (OR: 8.3, 95% CI: 0.7-94.7 from [37] and OR 1.6, 95% CI: 0.5-4.8 from [38]). However, a large study of acute lymphoblastic leukemia found no association with maternal occupational exposure to PCE during pregnancy ([39], and a small study of childhood cancer found no association among residents’ exposure to volatile organic compounds such as TCE and PCE through soil vapor intrusion into buildings [40].

In addition, even though neurotoxicity is considered a sensitive endpoint for assessing adverse health impacts of PCE exposure, the published literature on the risk of epilepsy following solvent exposure is sparse. To the best of our knowledge, only one epidemiological study has investigated the relationship between epilepsy and occupational exposure to organic solvents [41]. This Swedish case-control study found a 1.7-fold increased risk of epilepsy among individuals with any probable exposure to unspecified solvents (p = 0.20) and a 4.4-fold increased risk among individuals with probable heavy solvent exposure (p = 0.05). These associations are supported by several case reports documenting the occurrence of seizures following occupational or recreational exposure to mixed solvents [42,43], trichloroethylene [44], and glues composed of toluene and xylene [45-48] among previously healthy adults and adolescents.

In summary, the results of the present study suggest that the risk of certain types of cancer such as cervical cancer and epilepsy may be increased among adults exposed to PCE during gestation and early childhood. These findings should be interpreted cautiously because of the study limitations and confirmed in follow-up investigations of similarly exposed populations that include medical confirmation of self-reported diseases. The study population should also be monitored periodically for any changes in disease risk in this relatively young population. Since PCE remains a commonly used commercial solvent that exposes workers and consumers and frequently contaminates drinking water, it is important to determine the long-term impact of early life exposure.

## Conclusions

While adult exposure to PCE is known to have numerous toxic effects, there is little information on the long-term impact of prenatal and early childhood exposure. We undertook a retrospective cohort study to examine whether early life exposure to PCE-contaminated drinking water influenced the risk of a variety of chronic conditions among adults who were born in the Cape Cod area of Massachusetts. The results suggest that the risk of certain types of cancer such as cervical cancer and epilepsy may be increased among adults following PCE exposure during gestation and early childhood. No increases in the risk of obesity, diabetes, cardiovascular disease, hypertension, color blindness and other visual abnormalities were observed. These findings should be interpreted cautiously because of the study limitations and confirmed in follow-up investigations of similarly exposed populations that include medical confirmation of disease. The relatively young study population should also be monitored periodically for any changes in disease risk.

## Abbreviations

CI: Confidence Interval
DEP: Department of Environmental Protection
GEE: Generalized estimating equation
PCE: Tetrachloroethylene
RR: Risk Ratio
VL/AC: vinyl-lined asbestos-cement
